# CEACAM1 participation in breast cancer progression

**DOI:** 10.1002/1878-0261.70306

**Published:** 2026-07-10

**Authors:** Mykola Lyndin, Irina Kube‐Golovin, Anatolii Romaniuk, Gunther Wennemuth

**Affiliations:** ^1^ Department of Anatomy University Hospital Essen Essen Germany; ^2^ Department of Pathology, Academic and Research Medical Institute Sumy State University Sumy Ukraine

**Keywords:** breast cancer, CEACAM1, CEACAM1‐4L, cell proliferation, tumor progression

## Abstract

Carcinoembryonic antigen‐related cell adhesion molecule 1 (CEACAM1) is widely expressed in human cells and undergoes marked alterations during tumorigenesis. However, its functional significance in invasive breast cancer (BC) remains insufficiently understood. In this study, CEACAM1 expression patterns in invasive BC tissues were analyzed and correlated with the proliferative index (PI) of the tumor cells. To assess isoform‐specific functional effects and identify CEACAM1‐responsive genes involved in key proliferation‐associated pathways, MCF‐7 cells were transfected with either *CEACAM1* short cytoplasmic domain isoform (CEACAM1‐4S) or *CEACAM1* long cytoplasmic domain isoform (CEACAM1‐4L). BC tissues exhibited substantial heterogeneity in CEACAM1 expression, which correlated with the PI of the tumor cells. Functional *in vitro* studies demonstrated that the CEACAM1‐4L isoform exclusively suppresses cell proliferation. Transcriptome and protein‐level analyses revealed that CEACAM1‐4L regulates the expression of key mediators involved in cell cycle control, apoptosis, extracellular matrix organization, and growth factor‐dependent signaling pathways. These findings highlight the biological significance of CEACAM1‐4L isoform in BC, its potential diagnostic relevance for patient stratification, as well as its possible consideration in future therapeutic approaches.

## Graphical abstract

In invasive breast cancer (BC), CEACAM1 shifts from an apical to a uniform membranous/cytoplasmic pattern, or is lost, as tumors dedifferentiate, inversely tracking the Ki‐67 proliferative index. In MCF‐7 cells, only CEACAM1‐4L suppresses proliferation, repressing cell cycle and growth factor genes. CEACAM1‐4L acts subsequently as a gatekeeper of proliferation in breast cancer. Graphical abstract created with BioRender software.

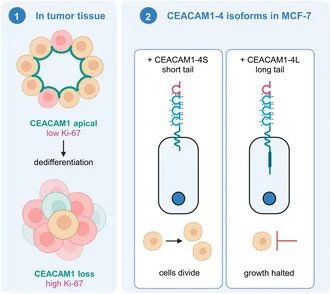

**Abbreviations**
Ab: Antibody
BC: breast cancer
BSA: Bovine serum albumin
CEACAM1: Carcinoembryonic antigen‐related cell adhesion molecule 1
CEACAM1‐4L: CEACAM1 long cytoplasmic domain isoform
CEACAM1‐4S: CEACAM1 short cytoplasmic domain isoform
DAPI: 4′,6‐diamidino‐2‐phenylindole
ER: Estrogen receptor
HER2: Human epidermal growth factor receptor 2
Ki‐67: Marker of proliferation Ki‐67 protein
mAb: Monoclonal antibody
PBS: Phosphate‐buffered saline
PI: Proliferative index
PR: Progesterone receptor
VeCo: Vector control (empty vector control)

## 1 Introduction

Breast cancer (BC) is the most frequently diagnosed cancer type and the leading cause of cancer‐related mortality among women worldwide, with annual incidence rates increasing by approximately 1–5% [1]. Recent projections indicate that by 2040, the global burden of BC is expected to rise to over three million new cases and one million deaths annually [2]. Timely diagnosis and personalized treatment are crucial for the effective management of BC [3]. The main diagnostic methods used for BC include imaging, tissue sampling, and molecular/pathological analyses. Currently, the immunohistochemical evaluation of BC mainly relies on the assessment of estrogen receptor (ER), progesterone receptor (PR), human epidermal growth factor receptor 2 (HER2), and the marker of proliferation Ki‐67 protein (Ki‐67). Although these markers provide important diagnostic and prognostic information, their predictive accuracy and ability to capture tumor heterogeneity remain limited, which affects the accuracy of patient management [4].

A highly promising marker for BC progression may be carcinoembryonic antigen‐related cell adhesion molecule 1 (CEACAM1), a member of the CEACAM family, which is expressed in a wide range of epithelial cells and leukocyte subtypes [5, 6]. This highly glycosylated protein is expressed in both membrane‐bound and soluble isoforms and is involved in diverse biological processes, including intercellular communication, intracellular signaling, regulation of cell survival and apoptosis, cell migration, modulation of immune responses, and pathogen binding [5, 6, 7, 8, 9]. CEACAM1 is expressed on the apical pole of breast luminal and glandular epithelium [10, 11, 12]. During mammary morphogenesis, two isoforms of CEACAM1 (CEACAM1 short cytoplasmic domain isoform (CEACAM1‐4S) and CEACAM1 long cytoplasmic domain isoform (CEACAM1‐4L)) are involved in cell polarization and lumen formation [12, 13, 14]. Additionally, by being located on the surface of extracellular vesicles secreted from the breast epithelium into milk, CEACAM1 may play a crucial role in lipid absorption in the infant gastrointestinal tract [12].

During neoplastic transformation and BC progression, CEACAM1 expression can become significantly dysregulated [11, 12, 15, 16, 17, 18]. Although CEACAM1 is consistently expressed in the surrounding intact tissues, the proportion of CEACAM1‐positive BC cases varies between 56% and 100% [11, 12, 15, 16, 17]. Furthermore, carcinoma dedifferentiation is associated with altered intracellular distribution of CEACAM1, characterized by the appearance of both uniform membranous and cytoplasmic patterns [10, 11, 12]. Compared with adjacent tissues, CEACAM1 expression in BC can exhibit both down‐regulation [10, 11, 12, 13, 14, 15, 16, 17] and up‐regulation [10]. Conversely, other studies report no difference in CEACAM1 expression between normal and tumor breast tissues, suggesting that the primary distinction lies in the redistribution of CEACAM1 from the apical membranous to a uniform membranous and cytoplasmic pattern [11, 18]. Despite the established correlation between CEACAM1 expression and the histological subtype of BC [12, 16], reported associations with metastasis [16] and ER/PR expression [10], its relationship with variables such as patient age, differentiation status, and HER2 or Ki‐67 expression remains unclear [10, 15, 16]. Nonetheless, CEACAM1 is involved in cell cycle regulation through its interactions with Rb, Rb2, and Id‐1 proteins in breast tissue [11, 15].

Throughout the appearance and progression of BC, variations in the CEACAM1‐L/S ratio are observed, suggesting that altered splicing of CEACAM1 plays an important role in tumorigenesis [19, 20]. The altered CEACAM1‐S/L ratio may underlie the variability in CEACAM1 effects on the functioning of both non‐malignant and cancer cells, regardless of whether its expression in tumor tissues is normal or dysregulated [5]. Kirshner J. et al. reported that restoration of CEACAM1‐4S in the CEACAM1‐negative MCF‐7 mammary carcinoma cell line is sufficient to reestablish normal cellular morphology [20].

This study investigates the functional roles of CEACAM1 isoforms and their potential diagnostic significance in invasive BC tissues, as well as their association with the proliferative index (PI) of carcinoma cells. We showed that cells within malignant breast tissue exhibit variability in both the expression levels and intracellular distribution of CEACAM1, which exerts a suppressive effect on cell proliferation. By transfecting MCF‐7 breast cancer cells with CEACAM1‐4S and CEACAM1‐4L, we demonstrated that only the CEACAM1‐4L isoform has an inhibitory effect on cell proliferation by dysregulating the expression of multiple genes involved in cell cycle–related and proliferation‐associated pathways.

## 2 Materials and methods

### 2.1 Tissue collection

Human postoperative non‐malignant (*n* = 10) and tumor (*n* = 30) breast tissue samples were obtained from patients treated at the Sumy Regional Oncology Center between 2019 and 2021 (Sumy, Ukraine). All patients were provided with written information about the study by their treating physicians and gave written informed consent for tissue investigation. These consent forms are stored in the patients' inpatient health records. The study was conducted in accordance with the Declaration of Helsinki. The experimental study protocol and the study itself were approved by the Bioethics Commission of the Academic and Research Medical Institute of Sumy State University (approval no. 20, dated September 21, 2018). The studies were conducted in accordance with the local legislation and institutional requirements. In this study, we focused exclusively on invasive carcinoma of no special type (previously referred to as invasive ductal carcinoma), the most prevalent BC subtype, which more frequently expresses CEACAM1 [11, 12, 16]. This selection allowed us to minimize the confounding effects of different histological tumor subtypes on the results. Tumor tissues were classified according to WHO guidelines and stratified according to histological grades of malignancy (G1, G2, and G3), as well as molecular subtypes defined by immunohistochemical assessment of ER, PR, HER2, and Ki‐67 expression. All diagnoses were independently confirmed by at least two pathologists.

### 2.2 Immunohistochemistry

#### 2.2.1 3,3′‐diaminobenzidine staining

Serial 4 μm sections were prepared from paraffin‐embedded tissue and mounted on 3‐aminopropyltriethoxysilane‐coated slides. After drying at 50 °C overnight, the sections were deparaffinized and rehydrated through graded alcohols and xylene. Heat‐induced antigen retrieval was performed in 0.01 m sodium citrate buffer at 97 °C for 30 min. Endogenous peroxidase activity was blocked with 3% H_2_O_2_ for 5 min, followed by phosphate‐buffered saline (PBS) washing. Nonspecific antibody (Ab) binding was blocked with 1% bovine serum albumin (BSA) in PBS for 1 h. Sections were incubated overnight at 4 °C with mouse anti‐CEACAM1 (clone C5‐1X/8; LeukoCom, Germany; 10 μg/mL), mouse anti‐Ki‐67 (clone MIB‐1; Dako, USA; 1:100 dilution), rabbit anti‐ER alpha (clone SP1; Abcam, UK; 1:200 dilution), rabbit anti‐PR (clone SP2; Abcam, UK; 1:400 dilution) monoclonal antibodies (mAb), and rabbit anti‐HER2 (Dako, USA; 1:150 dilution) polyclonal Ab, with isotype‐matched Ig as negative control. After PBS washing, sections were incubated with biotinylated rabbit anti‐mouse or swine anti‐rabbit secondary Ab (Dako, USA) for 1 h at room temperature, followed by VECTASTAIN ABC reagent (Vector Laboratories, USA) for 30 min. Signals were visualized using 3,3′‐diaminobenzidine, and sections were counterstained with hematoxylin. The sections were subsequently dehydrated through graded alcohols and xylene and mounted using Xylene Substitute Mountant (Thermo Fisher Scientific, USA).

#### 2.2.2 Immunofluorescence staining

The initial steps of immunofluorescence staining, including secondary Ab incubation, followed the previously described protocol in section 2.1. Cy3‐streptavidin (Jackson ImmunoResearch, UK) was used to visualize biotinylated secondary Ab, while cell nuclei were stained with 4′,6‐diamidino‐2‐phenylindole (DAPI) (Serva, Germany). Both reagents were diluted 1:200 in 0.5% BSA/PBS and applied simultaneously as a single staining cocktail. Sections were washed twice in PBS after each staining step and mounted with Fluoromount‐G® (SouthernBiotech, USA).

For double immunofluorescence staining, 4 μm sections of paraffin‐embedded tissues were treated after deparaffinization and rehydration with the MaxBlock® Autofluorescence Reducing Reagent Kit (Dianova, Switzerland) to minimize autofluorescence, followed by washes in 60% ethanol and distilled water. Heat‐induced antigen retrieval, blocking of nonspecific Ab binding, and incubation with anti‐CEACAM1 mAb and biotinylated secondary Ab were performed as previously described. Cy3‐conjugated streptavidin was then applied, followed by overnight incubation at 4 °C with rabbit anti‐Ki‐67 polyclonal Ab (Dako, USA). Mouse and rabbit IgG served as isotype controls. Donkey anti‐rabbit Alexa 488 and DAPI were both diluted 1:200 in 0.5% BSA/PBS and used to detect receptor‐positive signals and cell nuclei. Prior to mounting with Fluoromount‐G® (SouthernBiotech, USA), the MaxBlock® Post‐Detection Conditioner Kit was applied.

Signal detection was conducted using a Nikon Eclipse Ni‐E microscope equipped with a Ri2 camera and NIS‐Elements software version 5.30.02 (Nikon, Germany).

### 2.3 Cell culture

The human BC cell line MCF‐7 (RRID: CVCL_0031) was purchased from the American Type Culture Collection (ATCC/LGC Standards GmbH, Germany) and authenticated by short tandem repeat (STR) profiling within the last 3 years. The cells were maintained in Dulbecco's modified Eagle medium (Gibco, USA) supplemented with 10% heat‐inactivated fetal calf serum (Gibco, USA), 2 mm L‐glutamine (Gibco, USA), 100 U/mL penicillin (Gibco, USA), and 100 μg/mL streptomycin (Gibco, USA) at 37 °C in a humidified 5% CO_2_ atmosphere. The cells were mycoplasma‐free as verified by regular testing.

### 2.4 Transfection

MCF‐7 cells (2 × 10^5^ cells/well in a six‐well plate) were seeded and incubated at 37 °C in a humidified 5% CO_2_ atmosphere. When the cells reached 70% confluence, the culture medium was replaced, and the cells were stably transfected with the pcDNA3.1 neo(−) plasmid encoding either the human CEACAM1‐4L or CEACAM1‐4S isoform, using the jetPRIME® transfection reagent (Polyplus‐transfection, France; Cat. No. 101000046) according to the manufacturer's instructions. Cells transfected with the empty pcDNA3.1 neo(−) vector served as a vector control (VeCo). Two days post‐transfection, the cells were transferred into a 100 × 20 mm cell culture dish (Greiner Bio‐One, Austria) followed by the addition of Dulbecco's modified Eagle medium (Gibco, USA) supplemented with 10% heat‐inactivated fetal calf serum (Gibco, USA), 2 mm L‐glutamine (Gibco, USA), and 1 mg/mL of G418 sulfate solution (Capricorn, Switzerland). The culture medium was replaced every two days to eliminate non‐transfected cells lacking the resistance gene. Surviving transfected cells formed colonies, which were selectively picked and subcloned by serial dilution (Corning, Cell Cloning by Serial Dilution in 96 Well Plates Protocol). The clones were analyzed by flow cytometry, and CEACAM1‐positive clones were expanded for subsequent experiments.

### 2.5 Flow cytometry

Indicated MCF‐7 cells (5 × 10^5^) were labeled with anti‐CEACAM1 mAb (clone B3‐17; LeukoCom, Germany), diluted in 3% FCS/PBS, for 1 h, followed by washing with 3% FCS/PBS. Subsequently, the cells were incubated with FITC‐conjugated anti‐mouse F(ab')_2_ secondary Ab (Dianova, Germany) for 30 min at 4 °C. Isotype‐matched Ig mAb was used to determine background fluorescence. Stained cell samples were analyzed using a MACSQuant10 flow cytometer (Miltenyi Biotec, Germany), with data processed via MACS Quantify Software 2.11 (Miltenyi Biotec, Germany). The dead cells, identified by propidium iodide staining (5 μg/mL), were excluded from the analysis.

### 2.6 Polymerase chain reaction (PCR)

#### 2.6.1 mRNA extraction

Total mRNA was extracted using the RNeasy Mini Kit (Qiagen, Germany, Cat. No. 74106) according to the manufacturer's instructions. The concentration and purity of isolated mRNA were determined with a NanoPhotometer® NP80 spectrophotometer (Implen, Germany), and mRNA integrity was verified by agarose gel electrophoresis through visual assessment of the 28S and 18S rRNA bands. Complementary DNA was synthesized using the High‐Capacity cDNA Reverse Transcription Kit (Applied Biosystems, Germany, Cat. No. 4368814).

#### 2.6.2 Reverse transcription polymerase chain reaction (RT‐PCR)

RT‐PCR was performed using a C1000 Touch® Thermal Cycler (Bio‐Rad, Germany) with 2.5 ng of complementary DNA, 0.5 μm of each forward and reverse primer (Table S1), 0.2 U of GoTaq® DNA Polymerase, and 5 × GoTaq® reaction buffer (Promega, USA). The thermal cycling conditions were as follows: initial denaturation at 95 °C for 5 min, 44 cycles of denaturation at 95 °C for 30 s, annealing at 60 °C for 45 s, and extension at 72 °C for 45 s, followed by a final extension at 72 °C for 5 min. PCR products were visualized by agarose gel electrophoresis following GelRed staining (1:20000) and imaged using a ChemiDoc® Touch Imaging System (Bio‐Rad, Germany).

#### 2.6.3 Quantitative reverse transcription polymerase chain reaction (qRT‐PCR)

qRT‐PCR was performed using the qTOWER^3^ thermal cycler (Analytik Jena, Germany) with specific primers (Table S1) and 5 × EvaGreen® QPCR‐Mix II (ROX) (Bio‐Budget, Germany). The thermal cycling conditions were as follows: initial denaturation at 95 °C for 15 min, followed by 45 cycles of denaturation at 95 °C for 15 s, annealing at 58 °C for 30 s, and extension at 72 °C for 30 s. A melting curve analysis was performed to verify amplification specificity. Relative mRNA expression levels were calculated using the 2^‐ΔΔCt^ method, with 18S and GAPDH serving as housekeeping genes.

### 2.7 RNAseq analysis

The concentration and quality of total RNA were assessed using a Qubit fluorometer (Invitrogen, USA) and an Agilent Bioanalyzer with RNA Nano or Pico chips (Agilent Technologies, USA). RNA libraries were prepared using the Lexogen QuantSeq 3’ mRNA‐Seq V2 Library Prep Kit with UDI and the UMI Second Strand Synthesis Module for QuantSeq FWD (Lexogen, Austria). Sequencing was performed on a NextSeq 2000 system (Illumina, USA). Raw sequencing reads were trimmed using TrimGalore (Babraham Bioinformatics, UK) and aligned to the human reference genome (hg38) with HISAT2 (Johns Hopkins University, Baltimore, MD, USA). Genes expressed in only one sample or with a mean expression below five were excluded from further analysis. All experiments were performed using biological triplicates (*n* = 3 per group). Statistical analysis and data visualization were performed in R (v. 4.5.1; R Foundation for Statistical Computing, Austria) using the packages DESeq2, ComplexHeatmap (v. 2.18.0), umap (v. 0.2.8.0), fgsea, and EnhancedVolcano (v. 1.14.0). Differentially expressed genes were selected based on statistical significance and expression fold‐change thresholds (adjusted *P*‐value [padj] < 0.05 and |log_2_FC| > 1.0). Heatmaps and volcano plots were generated to visualize gene expression patterns and differentially expressed genes across experimental groups.

### 2.8 Cell block immunocytochemistry

3 × 10^7^ cells were transferred into 15 mL Falcon tubes, centrifuged at 1200 rpm for 3 min at room temperature, and the resulting pellets were fixed in 3.7% formaldehyde for 4 h at room temperature. Following fixation, the cell pellets were washed three times with tap water and then mixed with 2% pre‐warmed agarose. After cooling at room temperature, the samples were fixed, dehydrated, and embedded in paraffin using the HistoCore PEARL tissue processor (Leica, Germany). Subsequently, serial 4 μm sections were prepared from the paraffin‐embedded cells and mounted on 3‐aminopropyltriethoxysilane‐coated slides. All subsequent steps of the immunocytochemistry analysis were analogous to the immunofluorescence staining of tissues described above.

### 2.9 Western blotting

Cells were lysed in RIPA buffer (1% Triton X‐100, 1% sodium deoxycholate, 0.1% SDS, 150 mm NaCl, 2 mm EDTA, 50 mm sodium fluoride) supplemented with a protease inhibitor cocktail set III (Merck Millipore, Germany) and PhosSTOP phosphatase inhibitor cocktail (Roche, Germany), using a Bioruptor® Pico sonicator (Diagenode, USA). Protein concentrations were determined with the Pierce® BCA Protein Assay Kit (Thermo Fisher Scientific, Germany, Cat. No. 23227) according to the manufacturer's instructions. The protein samples were mixed with Laemmli sample buffer (62.5 mm Tris–HCl, 2% SDS, 25% glycerol, 0.01% bromophenol blue, and 5% β‐mercaptoethanol) and denatured at 95 °C for 5 min. Denaturated proteins were separated by electrophoresis on 12% gels using the TGX Stain‐free FastCast Acrylamide Kit (Bio‐Rad, USA). Separated proteins were transferred by semi‐dry blotting on the Trans‐Blot Turbo PVDF membrane using the Trans‐Blot Turbo Transfer System (Bio‐Rad, USA). After blocking with 5% non‐fat dry milk under gentle shaking, membranes were incubated with mouse anti‐CEACAM1 mAb (clone B3‐17; LeukoCom, Germany), mouse anti‐Rb mAb (clone 4H1; Cell Signaling Technology, USA), and rabbit anti‐ERBB4 recombinant Ab (bsm‐61882R; Bioss Antibodies, USA), followed by HRP‐conjugated goat anti‐mouse (Thermo Fisher Scientific, USA) or goat anti‐rabbit (Jackson ImmunoResearch, USA) secondary Ab. Protein lysates from human CEACAM1‐transfected CHO cells (hCC1‐CHO) were used as a positive control. To confirm equal protein loading, membranes were probed with rabbit anti‐GAPDH mAb (clone 14C10; Cell Signaling Technology, USA), followed by HRP‐conjugated goat anti‐rabbit secondary Ab. Protein bands were visualized using the Clarity® Western ECL Substrate and detected with the ChemiDoc® Touch Imaging System (Bio‐Rad, Germany).

### 2.10 Gap closing assay

MCF‐7 cells (7 × 10^4^ cells per well) were seeded into Ibidi chambers (Culture‐Inserts 2 Well for self‐insertion, Ibidi GmbH, Germany) placed in a 24‐well plate (Greiner Bio‐One, Germany) and incubated overnight. The following day, inserts were removed to create a cell‐free gap, and the remaining cell patches were washed with PBS before adding 2 mL of medium per well. Time‐lapse imaging was performed with image acquisition every 30 min over a total period of 48 h. The gaps (*n* = 8/group) were monitored and the binary fraction area was calculated using NIS‐Elements BR Analysis 5.42.06 software (Nikon, Germany).

### 2.11 Colony forming assay

MCF‐7 cells were seeded in 6‐well plates (1 × 10^3^ cells per well) and cultured in complete growth medium under standard conditions (37 °C, 5% CO_2_) for 10 days. The medium was replaced every 3–4 days. At the end of the incubation period, colonies were gently washed twice with PBS, fixed with methanol/acetic acid (7:1, v/v) for 5 min, and stained with 0.5% crystal violet solution (prepared in 25% methanol) for 25 min. Excess dye was removed by washing with distilled water, and plates were air‐dried. Wells were photographed using the Fujifilm LAS‐3000 gel documentation system (Kleve, Germany), and the density of colonies was calculated as color units using ImageJ 1.53 t software.

### 2.12 5‐Bromo‐2′‐deoxyuridine (BrdU) assay

The cell proliferation was assessed using the BrdU (colorimetric) assay kit (Roche, Germany, Cat. No. 11647229001) according to the manufacturer's protocol. The signal intensity was measured in triplicates using a microtiter plate reader CLARIOstar Plus (BMG LABTECH, Germany).

### 2.13 Statistics

All quantitative data were analyzed and graphed using GraphPad Prism version 9 (GraphPad Software, San Diego, CA, USA). Data are presented as mean ± SD, unless indicated otherwise. Comparisons among three groups were performed using one‐way ANOVA followed by Bonferroni *post hoc* test, while pairwise comparisons were analyzed using Student's *t*‐test. Pearson's correlation analysis was applied to evaluate correlations between variables. qRT‐PCR expression data were analyzed using anti‐logarithmic data. Statistical significance was defined as *P < 0.05.

## 3 Results

### 3.1 CEACAM1 expression in breast tissues

CEACAM1 immunoreaction was observed on the apical surface of luminal epithelial cells throughout all regions of non‐malignant breast glandular‐ductal structures (Fig. 1A). Although the CEACAM1 staining intensity varied, its distribution on the cell surfaces remained almost uninterrupted. Some intra‐ and extravascular leukocytes, as well as the endothelium of isolated vessels, exhibited CEACAM1 positivity, serving as positive internal controls (Fig. 1B). Simultaneously, secreted and detached CEACAM1‐positive vesicles were observed in the lumens of the mammary gland ducts (Fig. 1B). In contrast, apocrine epithelial cells showed no CEACAM1 immunoreaction.

**Fig. 1:**
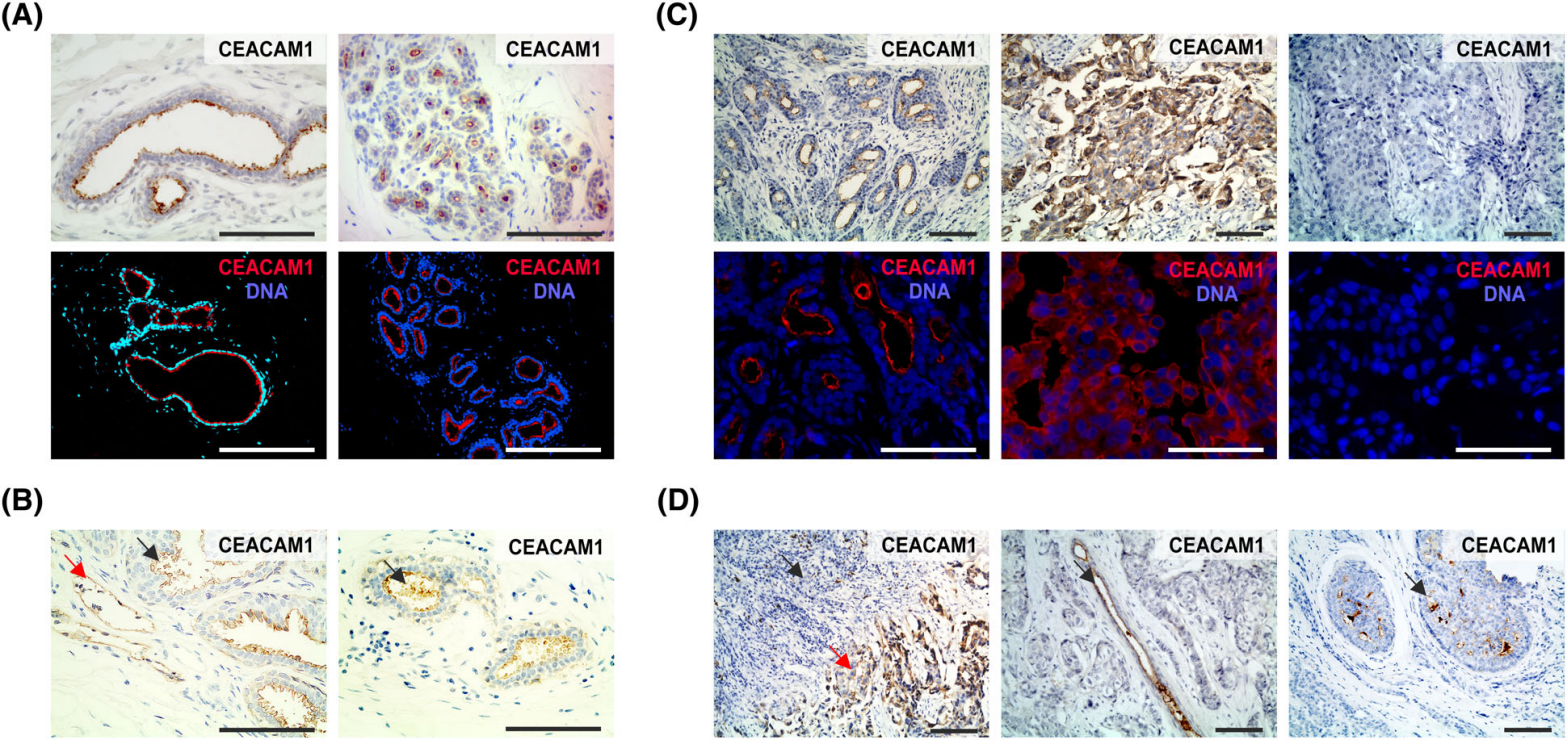
CEACAM1 expression in non‐malignant and tumor breast tissues. (A) Apical distribution of CEACAM1 in the ducts (left panel) and glands (right panel) of the non‐malignant mammary gland was detected by both immunohistochemical (upper panel) and immunofluorescence (lower panel) investigations. Representative microscopic images from 10 cases. Scale bar = 100 μm. (B) In addition to apical distribution in the epithelium, CEACAM1‐positive vesicles are present in the lumens of the mammary gland ducts (indicated by black arrows). CEACAM1‐positive vessels are observed in the surrounding tissues (indicated by a red arrow). Scale bar = 100 μm. (С) Immunohistochemical (upper panel) and immunofluorescence (lower panel) investigations reveal apical (left panel) and entire membranous‐cytoplasmic (middle panel) distribution of CEACAM, as well as its absence (right panel) in breast cancer tissues. Representative microscopic images from 30 cases. Scale bar = 100 μm. (D) Representative images show heterogeneous CEACAM1 expression in breast cancer tissues, with CEACAM1‐positive (red arrow) and CEACAM1‐negative (black arrow) tumor areas (left). CEACAM1 in normal breast structures (middle, indicated with an arrow) and foci of carcinoma *in situ* (right, indicated with an arrow) among CEACAM1‐negative tumor cells. Scale bar = 100 μm.

A highly heterogeneous distribution of CEACAM1 was observed within tumor tissues, displaying varied patterns among neoplastic cells (Table 1, Fig. 1C). Among all cases of invasive carcinoma of no special type, CEACAM1 immunoreaction was absent in only four samples, which were graded as G3. Well‐differentiated (G1) tumors exhibited CEACAM1 on the apical surface of tumor cells, which formed tubular structures.

**Table 1:** CEACAM1 in breast cancer lesions and their metastases.

Histological grade		CEACAM1 expression in primary tumors		Mts in l/n	CEACAM1 expression in metastasis	
		CEACAM1	Pattern		CEACAM1	Pattern
G1	20% (6/30)	100% (6/6)	A: 100% (6/6)	–	–	–
G2	40% (12/30)	100% (12/12)	A: 83% (10/12) A + M: 8.5% (1/12) A + C: 8.5% (1/12)	25% (3/12)	100% (3/3)	A + M: 33.3% (1/3) A + C: 33.3% (1/3) С: 33.3% (1/3)
G3	40% (12/30)	67% (8/12)	M: 50% (4/8) C: 25% (2/8) M + C: 12.5% (1/8) A + M + C: 12.5% (1/8)	67% (8/12)	62.5% (5/8)	M: 40% (2/5) M + C: 40% (2/5) C: 20% (1/5)

*Note:* Apical (A), cytoplasmic (C), and uniform membranous (M) pattern of CEACAM1. Mts in l/n—metastases in lymph nodes.

Tumor dedifferentiation was accompanied by changes in both histological architecture and the presence and distribution patterns of CEACAM1. On the one hand, the lumen's loss and the formation of trabecular‐solid structures were accompanied by a decrease and partial absence of CEACAM1 immunoreactivity in tumor cells. On the other hand, the progression of cellular atypia accompanying tumor dedifferentiation was characterized by the redistribution of CEACAM1 from the apical cell surface to the entire membrane and cytoplasm (Fig. 1C).

The tumor tissues often exhibited heterogeneous patterns of CEACAM1, with areas showing either positive or negative immunoreactivity, or combined patterns in CEACAM1‐positive cases. This phenotypic heterogeneity was evident both within individual cases and across different regions of the same tumor, complicating a definitive determination of the CEACAM1‐positive or CEACAM1‐negative status (Fig. 1D). In addition, normal intra‐ and peritumoral breast structures, as well as some other proliferative lesions, including foci of carcinoma *in situ*, which exhibited CEACAM1 in epithelial cells, were observed among malignant tumor cells (Fig. 1D). Similar to non‐malignant breast tissue, intravascular and interstitial leukocytes, as well as isolated vessels showing CEACAM1 signal, were identified among tumor cells and in peritumoral tissues (Fig. S1A).

It is noteworthy that during metastasis, the CEACAM1 phenotype of cancer cells also changed, affecting both the level and subcellular distribution of CEACAM1 (Table 1, Fig. S1В). Among 11 cases of BC with lymph node metastasis, six demonstrated consistent CEACAM1 expression in both primary and metastatic tumors. In five cases, changes in CEACAM1 expression were observed: in three cases, alterations in expression patterns were detected, while in the remaining two cases, one showed the emergence of CEACAM1 expression in metastatic tissue despite its absence in the primary tumor, and the other exhibited a loss of CEACAM1 expression in metastatic tissue that was present in the primary tumor.

### 3.2 The correlation between cellular CEACAM1 distribution and the PI in BC tissues

Analysis of ER, PR, and HER2 expression revealed no significant association with CEACAM1 expression patterns (*P* > 0.05), which was further confirmed by serial‐section analysis demonstrating that variability in CEACAM1 distribution within tumor tissues was not accompanied by corresponding changes in ER, PR, or HER2 expression (Fig. S2).

Furthermore, we found no significant difference in the PI, measured as the percentage of Ki‐67‐positive cells, between CEACAM1‐positive and CEACAM1‐negative BC cases (*P* > 0.05) (Fig. 2A, left panel). However, when CEACAM1‐positive cases were divided into two groups based on apical versus uniform membranous/cytoplasmic expression, tumors with exclusively apical CEACAM1 expression exhibited a lower PI compared to CEACAM1‐negative tissues (*P* = 0.047) and CEACAM1‐positive tumors with uniform membranous/cytoplasmic expression (*P* = 0.027) (Fig. 2A, middle panel). Analysis of serial sections and double immunohistochemical staining confirmed significant differences among the three tissue groups (Fig. 2A,B). Distinct fields of view lacking CEACAM1 expression exhibited a higher PI compared to tumors with apical (*P* < 0.0001) or uniform membranous/cytoplasmic CEACAM1 expression (*P* = 0.041), while the PI in tumors with uniform membranous/cytoplasmic expression was higher than in tumors with apical CEACAM1 expression (*P* = 0.013) (Fig. 2A, right panel). These findings were further confirmed by examining tumor regions with heterogeneous CEACAM1 expression, which demonstrated significant differences in the number of Ki‐67‐positive cells depending on the presence and intensity of CEACAM1 signal (Fig. 2C).

**Fig. 2:**
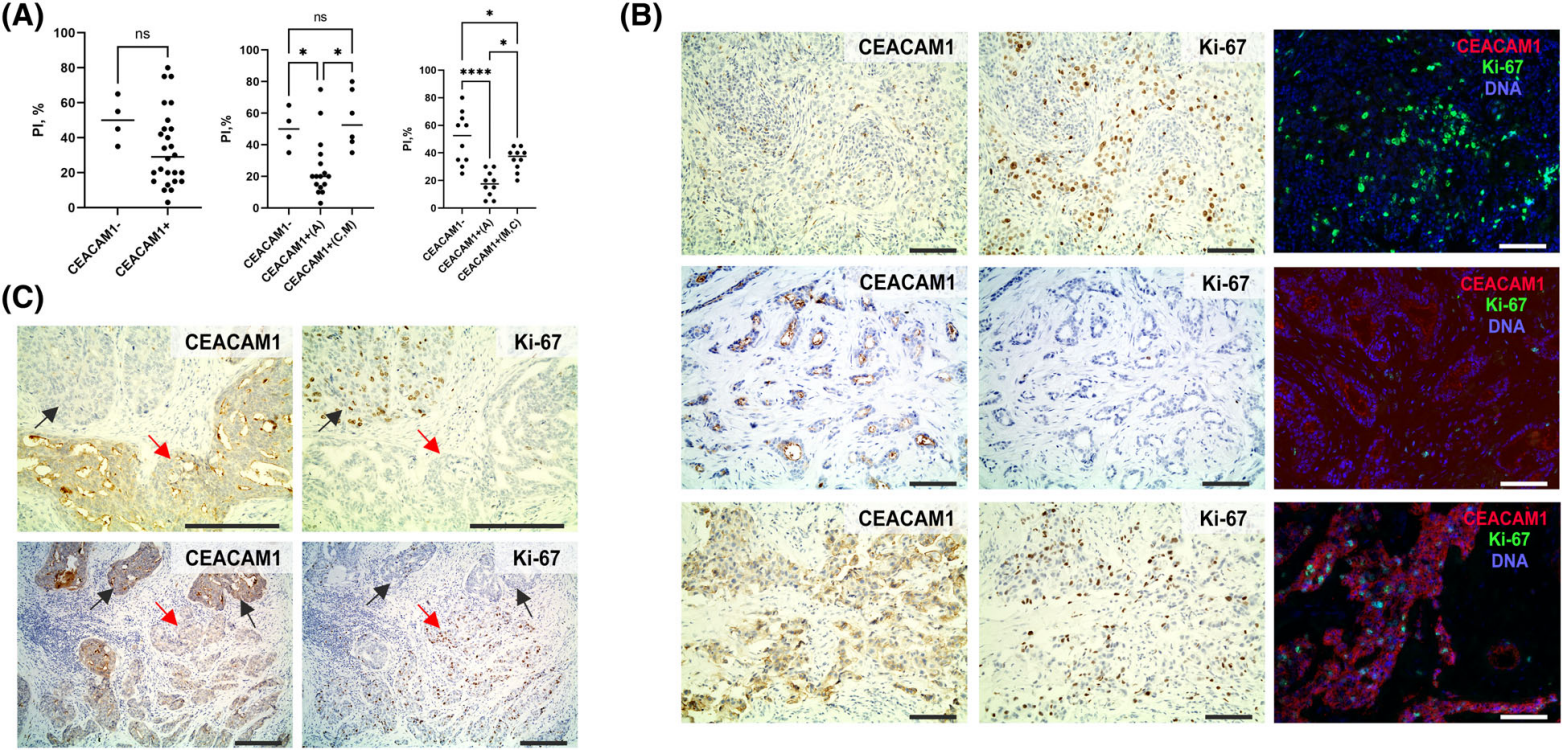
The correlation between CEACAM1 and the proliferative index in breast cancer tissues. (A) Comparative analysis of proliferative index (PI) among CEACAM1‐negative and CEACAM1‐positive tumor groups (left); among CEACAM1‐negative tumors and tumors with apical, as well as uniform membranous/cytoplasmic CEACAM1 distribution (middle); and within distinct visual fields with varying CEACAM1 distribution (right). Data are represented as mean ± SD; one‐way ANOVA followed by Bonferroni *post hoc* analysis, ns = not significant, *P < 0.05, *n* = 30. (B) Representative figures from 30 cases showing variations in PI in visual fields of CEACAM1‐negative tumors (upper panel), as well as in CEACAM1‐positive tumor regions with apical (middle panel) and uniform membranous/cytoplasmic distribution of CEACAM1 (lower panel). Scale bar = 100 μm. (С) Serial tissue sections demonstrating different PI in tumor foci with (indicated by a red arrow) and without (indicated by a black arrow) CEACAM1 (upper panel). The lower panel demonstrates the variations of PI in tumor regions with strong (indicated by a black arrow) and weak (indicated by a red arrow) CEACAM1 signal. Scale bar = 200 μm. Representative figures from 30 cases.

### 3.3 Effect of CEACAM1 on the proliferative activity of MCF‐7 cells

To assess the impact of CEACAM1 on the proliferative activity of BC cells, we transfected the MCF‐7 cells with plasmids encoding either human *CEACAM1‐4L* or *CEACAM1‐4S*. This generated three distinct cell lines: a vector control lacking CEACAM1 expression (VeCo), and two expressing either hCEACAM1‐4L (+CEACAM1‐4L) or hCEACAM1‐4S (+CEACAM1‐4S).

Successful transfection of the cells was confirmed by different methods. The absence of specific antibodies for the CEACAM1 L‐ and S‐isoforms limited our ability to confirm the +CEACAM1‐4L or + CEACAM1‐4S phenotype in MCF‐7 cells. Therefore, transcript‐level analysis was used to distinguish between the transfected CEACAM1 isoforms. mRNA expression was analyzed using reverse transcription polymerase chain reaction, followed by visualization of the amplicons by agarose gel electrophoresis (Fig. 3A). CEACAM1 protein expression was confirmed by flow cytometry (Fig. 3B). Western blot analysis verified the presence of CEACAM1 in whole‐cell lysates based on its molecular weight (Fig. 3C), whereas immunocytochemistry revealed CEACAM1 predominantly membranous, with partial cytoplasmic localization (Fig. 3D).

**Fig. 3:**
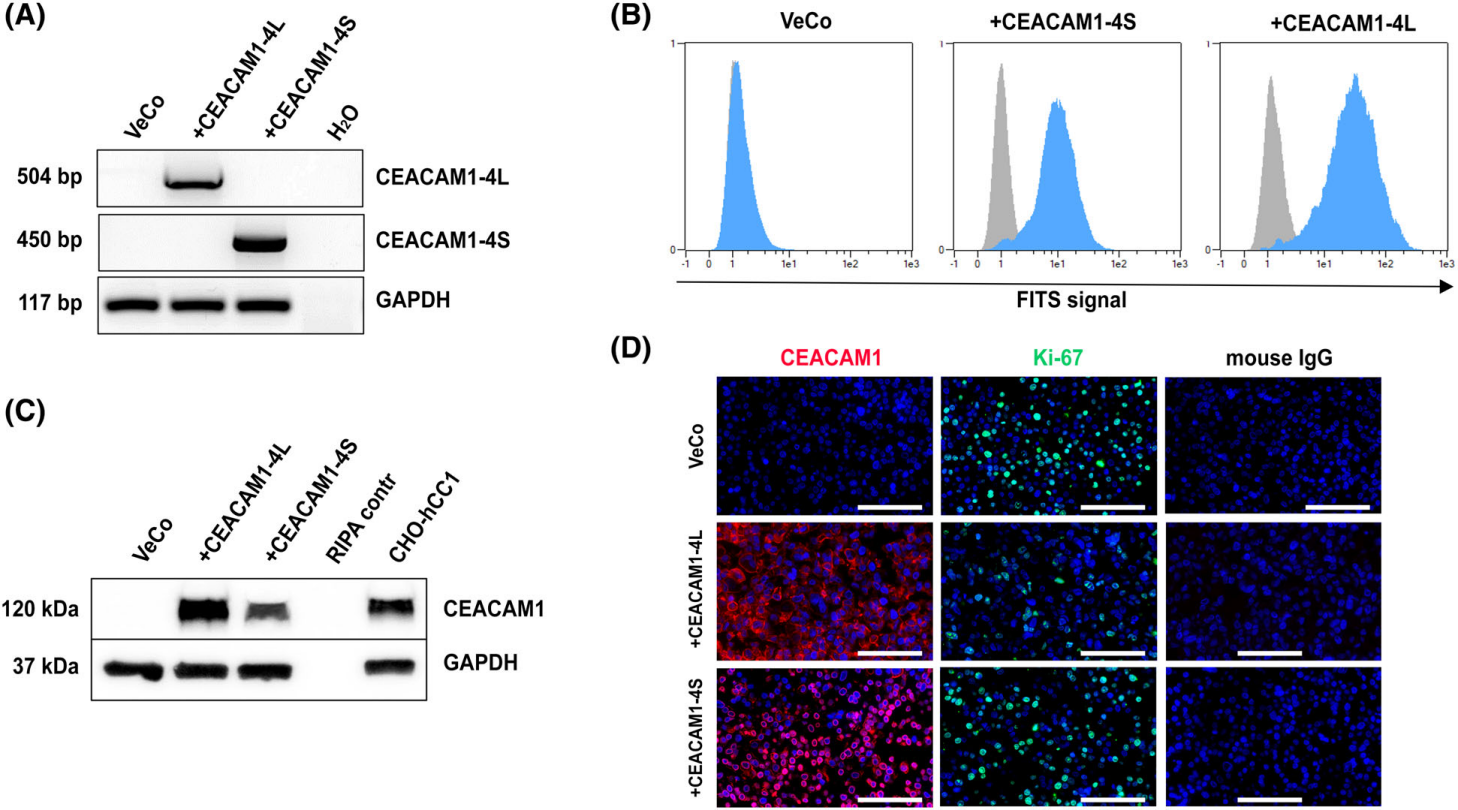
Successful *CEACAM1* transfection in MCF‐7 cells. (A) Representative agarose gel electrophoresis of RT‐PCR products using *GAPDH* as a reference gene and H_2_O as a non‐template control. Representative blot from *n* = 3. Exclusive detection of *CEACAM1‐4L* expression in +CEACAM1‐4L cells, and *CEACAM1‐4S* expression solely in +CEACAM1‐4S cells is shown. (B) Flow cytometry analysis shows positive fluorescence signals for CEACAM1 (B3‐17 mAb) on the surface of MCF‐7 cells, transfected with *hCEACAM1‐4L* and *hCEACAM1‐4S*. mAb stainings are indicated with a blue histogram, negative control staining (mouse IgG) is indicated with a gray histogram. (C) Western blot analysis of CEACAM1 expression in MCF‐7 cells. CEACAM1 (~ 120 kDa, B3‐17 mAb) was detected in +CEACAM1‐4L and + CEACAM1‐4S cells. Positive control: protein lysate from CHO cells transfected with human *CEACAM1* (CHO‐hCC1). GAPDH (~ 37 kDa) served as loading control. Representative blot from *n* = 3. (D) Cell block immunocytochemistry. CEACAM1 was detected in *CEACAM1‐4L*‐ and *CEACAM1‐4S*‐transfected MCF‐7 cells. Ki‐67 staining showed a reduced proportion of positive cells in +CEACAM1‐4L compared with VeCo and + CEACAM1‐4S. The fluorochrome DAPI visualizes DNA in the cell nuclei (blue). Negative control: mouse IgG. Scale bar = 100 μm.

In *CEACAM1‐4L*‐transfected MCF‐7 cells, the proliferative activity was significantly lower compared with both VeCo and + CEACAM1‐4S. After seeding an equal number of cells, a significantly lower cell count was observed in the +CEACAM1‐4L group following three days of cultivation (Fig. 4A). The anti‐proliferative role of CEACAM1‐4L was further confirmed by a range of functional assays. Specifically, a markedly lower incorporation of BrdU into newly synthesized DNA, reflecting slower cell division, was observed in +CEACAM1‐4L cells compared with both VeCo and + CEACAM1‐4S transfectants in the BrdU assay (Fig. 4B). In the gap closing assay, +CEACAM1‐4L cells exhibited slower gap closure compared with the other two groups (Fig. 4C), reflecting both reduced migratory capacity and decreased proliferative potential associated with CEACAM1‐4L expression. Finally, colony formation assays demonstrated a pronounced decrease in the clonogenic potential of +CEACAM1‐4L cells. Colonies formed by these cells were smaller and fewer in number compared with those formed by VeCo and + CEACAM1‐4S cells (Fig. 4D). In addition, quantification of Ki‐67–positive cells in cell block immunocytochemistry images further supported these findings. The PI of +CEACAM1‐4L cells was significantly lower than that of VeCo and + CEACAM1‐4S cells (Fig. 3C, Fig. S3). Although some variability was noted, differences between VeCo and + CEACAM1‐4S cells in functional assays and PI were not statistically significant.

**Fig. 4:**
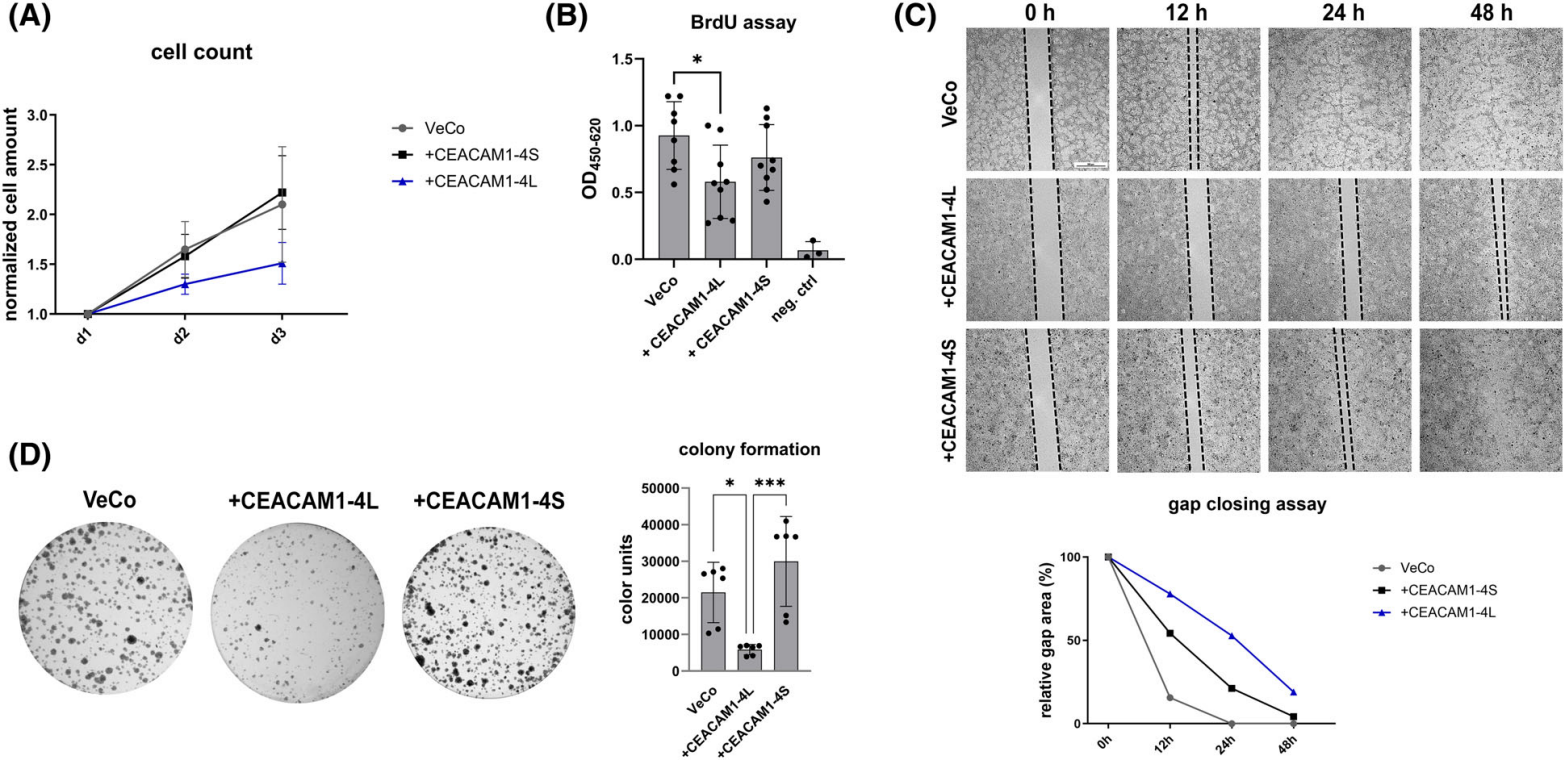
The effect of CEACAM1‐4S/L isoforms on the proliferative activity of MCF‐7 cells. (A) The graph displays the normalized cell amount on day 1, 2 and 3 of cultivation. A noticeable decline in the growth rate of *CEACAM1‐4L*‐transfected cells compared to VeCo and *CEACAM1‐4S*‐transfected cells is observed. Data are represented as mean ± SD, *n* = 3. (B) BrdU incorporation assay. Decreased proliferation rate in +CEACAM1‐4L cells compared to VeCo and + CEACAM1‐4S cells. Data are represented as mean ± SD, one‐way ANOVA followed by Bonferroni *post hoc* analysis, *P < 0.05, *n* = 3–9. (C) Gap closure assay. Representative images showing gap closure of VeCo, +CEACAM1‐4L, and + CEACAM1‐4S cells. Quantification reveals differences in relative gap area, indicating slower gap closure in +CEACAM1‐4L cells. Scale bar = 200 μm, *n* = 3. (D) Colony forming assay. Representative images of colony formation using VeCo, +CEACAM1‐4S, and + CEACAM1‐4L cells after seeding and growth for 10 days, followed by fixation and crystal violet staining. Quantification was performed by measuring the color units (right). Data are presented as mean ± SD; one‐way ANOVA followed by Bonferroni *post hoc* analysis, *P < 0.05, *n* = 6.

### 3.4 Isoform‐specific effects of CEACAM1‐4 on proliferation‐associated gene and protein expression in MCF‐7 cells

The above experiments demonstrated that introducing CEACAM1‐4L into MCF‐7 cells is associated with reduced proliferative activity, implying a potential function of this isoform in restraining cell cycle progression. To identify CEACAM1‐4‐responsive genes associated with BC cell biology, transcriptome sequencing (RNA‐Seq) was performed on the MCF‐7 transfectants: VeCo, +CEACAM1‐4L, and + CEACAM1‐4S. As shown in the heatmap (Fig. 5A), a substantial number of genes were up‐ or downregulated in +CEACAM1‐4L‐ and + CEACAM1‐4S cells compared with VeCo. Subsequently, we selected and analyzed a proliferation‐related gene set (human GOBP_EPITHELIAL_CELL_PROLIFERATION; M15437), which revealed that transfection of MCF‐7 cells resulted in significant dysregulation of 26 transcripts, exhibiting greater than twofold changes compared with VeCo cells (Fig. 5B, Table S2). These transcripts are implicated in multiple pathways that collectively influence cellular proliferation, including cell cycle regulation (*RB1, CCND2, NUPR1, STAT1*), hormone signaling (*PGR*), apoptosis and stress responses (*HTRA1, HMOX1, THBS1, NR4A1*), extracellular matrix organization and cell adhesion (*GJA1, CLDN1, PTPRK, APOA1, AGAP2*), growth factor signaling and transcriptional regulation (*IGFBP5, FGF18, TGFB2, AREG, ERBB4, JAK2, STAT6, FST, ERRFI1, EGR3, ETV4, OSR2*) [21, 22, 23, 24].

**Fig. 5:**
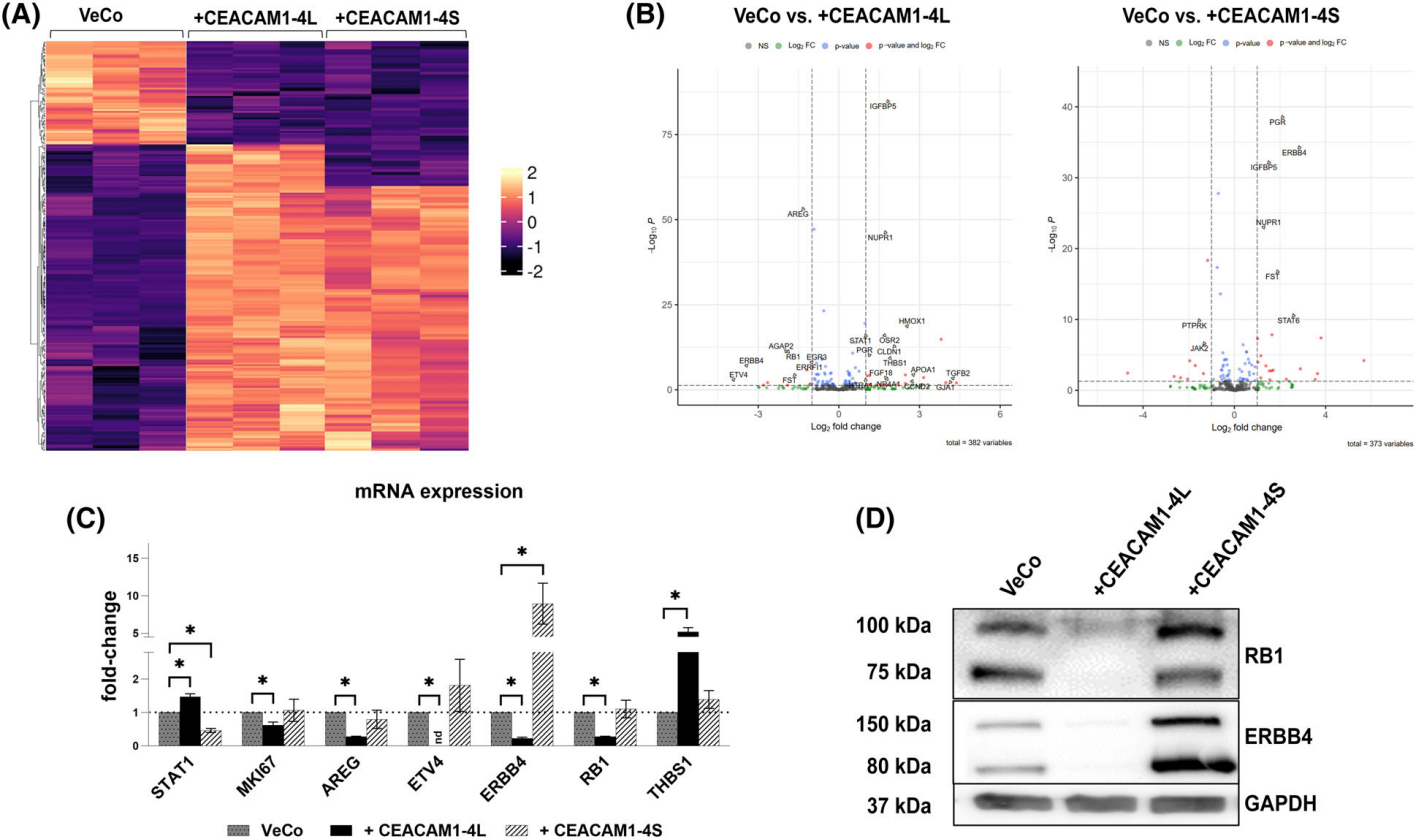
Effect of *CEACAM1‐4L/S* on proliferation‐associated genes and proteins in MCF‐7 cells. (A) Overview of RNA‐Seq results. The heatmap shows the gene expression in VeCo, +CEACAM1‐4S, and + CEACAM1‐4L transfectants (*n* = 3). The x‐axis represents individual samples, and the y‐axis represents genes. The color intensity corresponds to expression levels, with red indicating upregulated and blue indicating downregulated genes. (B) Volcano plots of RNA‐Seq analysis. Volcano plots showing differentially expressed genes within the proliferation‐related gene set in +CEACAM1‐4L (left) and + CEACAM1‐4S (right) cells compared to VeCo. Significantly dysregulated proliferation‐associated genes (above thresholds *P* < 0.05, log_2_FC > 1.0) are highlighted. (C) qRT‐PCR analysis of selected proliferation‐related genes. Transfection with *CEACAM1‐4L* and *CEACAM1‐4S* leads to significant dysregulation of *STAT1, MKI67, AREG, ETV4, ERBB4, RB1, and THBS1*. Relative mRNA expression levels were calculated using the 2^−^ΔΔCt method (fold‐change), with *18S* and *GAPDH* serving as housekeeping genes. The data are normalized to VeCo (dotted line, fold‐change = 1). Data are presented as mean ± SEM. One‐way ANOVA followed by Bonferroni *post hoc* test: *P < 0.05; nd = not detected, *n* = 3. (D) Western blot analysis. Representative immunoblots showing decreased expression of RB1 (~ 100 and ~ 75 kDa) and ERBB4 (~ 80 and ~ 150 kDa) in *CEACAM1‐4L* transfectants, and elevated ERBB4 expression in *CEACAM1‐4S* transfectants. GAPDH (~ 37 kDa) used as a loading control. Representative blot from *n* = 3.

To validate the expression variability observed in the RNA‐Seq analysis, we performed qRT‐PCR on a subset of proliferation‐related genes (*STAT1, AREG, ETV4, ERBB4, RB1*, and *THBS1*), which were selected based on two criteria: (i) the most significant differential expression in RNA‐Seq (lowest *P*‐values and highest log_2_FC), and (ii) the biological relevance of the encoded proteins in the context of BC. Specifically, *MKI67* (Ki‐67) was included as a widely accepted marker of proliferative activity, which also exhibited different expression in BC tissues. The variability observed in the qRT‐PCR (Fig. 5C) closely reflected the RNA‐Seq results, and reduced *MKI67* expression in *CEACAM1‐4L*‐transfected MCF‐7 cells corresponded to their decreased proliferative activity.

As shown by western blot analysis (Fig. 5D), the observed changes in mRNA expression were mirrored by corresponding alterations at the protein level. Specifically, the protein expression of ERBB4 and RB1 was reduced in +CEACAM1‐4L cells, while ERBB4 expression was increased in +CEACAM1‐4S cells. Together, these results validate the RNA‐Seq findings and demonstrate that CEACAM1‐4 influences the expression of key proliferation‐associated genes.

## 4 Discussion

The identification and validation of reliable tumor‐specific markers with diagnostic and prognostic value remain central challenges in both biomedical research and clinical practice, particularly given the limited success in achieving curative outcomes across most malignancies [25]. Investigating CEACAM1 expression, the most broadly expressed member of the CEACAM family in humans, holds significant potential for advancing our understanding of cancer biology, including BC. CEACAM1 plays a pivotal role in signaling pathways that regulate tumor growth and metastasis, making it a promising candidate for diagnostic and therapeutic strategies [24, 26, 27].

In line with previous studies [10, 11, 12], we confirmed that CEACAM1 is consistently expressed on the apical surface of luminal epithelial cells throughout normal mammary ductal and glandular structures. Given the apocrine secretory activity of luminal epithelium, CEACAM1 within aposomes can detach from parent cells and mediate long‐range intercellular signaling [12, 28]. This variability is reflected in differences in CEACAM1 accumulation at the apical cell surface, and consequently in signal intensity upon detection.

Tumor transformation alters CEACAM1 expression in neoplastic cells. According to our findings and earlier reports [11, 12, 15, 16, 17], in well‐differentiated tumors, where neoplastic cells retain the ability to form tubular‐glandular patterns, CEACAM1 remains localized at the apical pole of tumor cells. The disappearance of lumens and the transition of tumor cells to trabecular‐solid growth patterns, accompanying the dedifferentiation of malignancies, result in the loss of CEACAM1 expression. Another aspect of CEACAM1 dysregulation involves its uniform distribution throughout the cell membrane and cytoplasm in poorly differentiated tumors [10, 11, 12], which we observed in over 30% of BC cases. These findings further indicate that not only the absence of CEACAM1 expression, but also its cytoplasmic or complete membranous localization, can serve as indicators of malignancy in breast tissue, thereby representing a potentially valuable tool for the differential diagnosis. In contrast, apical CEACAM1 signal can be observed in both breast carcinomas and normal glandular structures. Potential CEACAM1 expression in intra‐tumoral leukocytes, vascular endothelium, normal breast epithelium, and non‐malignant lesions should always be taken into account during evaluation, as this cellular and structural heterogeneity may pose a challenge for the implementation of automated or artificial intelligence‐based image analysis systems.

Tumor cells rarely exhibited uniform CEACAM1 expression within the same case, instead displaying pronounced intra‐tumoral heterogeneity. This underscores the potential for dynamic changes in CEACAM1 status during tumor progression and throughout both local and distant dissemination. The concept of antigenic atypia may underlie these phenomena. It can manifest either through antigenic simplification with a reduction in antigens synthesized by normal cells, as observed in cases where CEACAM1 disappears or through antigenic reversion with the emergence of a uniform membranous and cytoplasmic distribution of CEACAM1, resembling the expression pattern of some CEACAMs in cells during fetal development [29].

Comparing CEACAM1 status in BC tissues with the PI, we initially, in line with previous studies [15, 16], did not detect any correlation. However, upon analyzing individual tumor fields with and without CEACAM1 expression and considering the specific expression patterns (apical versus uniform membranous/cytoplasmic), we found that CEACAM1‐positive tumor cells had a lower PI, which also varied depending on the subcellular distribution and intensity of the CEACAM1 signal. The variability of the PI in tumor cells depending on the CEACAM1 expression pattern may be related to post‐translational modifications of CEACAM1, leading to alterations in its functional activity [30].

This led us to conclude that CEACAM1 may exert an inhibitory effect on cell proliferation, which, in the context of BC, may be mediated through its interaction with other membrane receptors or with intracellular cell cycle regulators [11, 15, 31, 32]. To further support this observation, we generated MCF‐7 BC cell lines expressing either *CEACAM1‐4L* or *CEACAM1‐4S*. Cells expressing *CEACAM1‐4L* demonstrated reduced growth in multiple functional assays, including measurements of cell accumulation over defined cultivation intervals, gap closure, clonogenic capacity, and other proliferation‐related readouts, whereas no significant differences were observed between VeCo and CEACAM1‐4S expressing cells, confirming that only the CEACAM1‐4L isoform, and not CEACAM1‐4S, specifically reduces cellular proliferative activity [31, 33, 34]. To further substantiate the involvement of CEACAM1‐4L in regulating cell proliferation, we performed transcriptome profiling of the MCF‐7 transfectants and assessed the variability of proliferation‐associated gene expression. CEACAM1‐4L expressing cells exhibit a pronounced dysregulation of transcripts associated with proliferative capacity, including key regulators of cell‐cycle progression, apoptosis and cellular stress responses, extracellular matrix organization and adhesion, growth factor‐mediated signaling and transcriptional control [21, 22, 23] (Fig. 5B, Table S2). The biological meaningfulness of the mRNA‐level changes was further selectively examined and confirmed at the protein level. These results indicate that CEACAM1‐4L modulates both gene expression and the resulting protein‐level outputs within key regulatory pathways controlling BC cell proliferation.

Despite these findings, several aspects of the study should be considered when interpreting the results and their translational implications. Our data establish CEACAM1 localization patterns across breast tumor grades and their association with proliferation in a discovery cohort. We did not predefine a scoring rubric or perform blinded reproducibility and external validation analysis. Diagnostic performance metrics, interobserver agreement, and external validation cohorts were not included and will be necessary to support clinical implementation of CEACAM1 staining patterns.

Our functional analyses demonstrate an anti‐proliferative effect of CEACAM1‐4L in MCF‐7 cells. However, we did not resolve CEACAM1 isoform composition within human tumor regions exhibiting distinct CEACAM1 localization patterns. Due to the lack of isoform‐selective antibodies and the complexity of FFPE tissues, we could not directly attribute apical versus uniform/cytoplasmic CEACAM1 patterns to CEACAM1‐4L or CEACAM1‐4S *in situ*. Additionally, loss‐of‐function experiments (e.g., siRNA‐mediated knockdown or CRISPR/Cas9‐based gene editing) were not performed in this study, and the functional findings are therefore based on gain‐of‐function models. This should be addressed in future studies.

## 5 Conclusion

Our study identifies CEACAM1 as a potential biomarker associated with BC pathogenesis, reflecting its variability during tumor progression and dissemination. Immunohistochemical analysis of BC tissues and functional *in vitro* experiments demonstrate that CEACAM1, specifically the CEACAM1‐4L isoform, reduces BC cell proliferation through the modulation of key genes involved in cell cycle regulation, apoptosis, and growth factor‐related signaling pathways, which is supported by transcriptomic profiling and validation at both RNA and protein levels. Thus, the loss of CEACAM1‐4L and/or the shift of CEACAM1‐4S/L ratio in BC tissue may give a diagnostic and prognostic value for patient management. Furthermore, the cellular distribution of CEACAM1 isoforms and its functional relevance should be addressed in further studies. In addition, these findings suggest that CEACAM1 may be considered in future therapeutic strategies aimed at restraining BC proliferation.

## Conflict of interest

The authors declare no conflicts of interest. This research was conducted without any commercial or financial relationships that could be construed as a potential conflict of interest. Additionally, the funders played no role in the study's design, data collection, analysis, interpretation, manuscript writing, or the decision to publish the findings.

## Author contributions

Conception, ML, IK. Methodology, ML, IK. Formal analysis, ML, IK, AR, GW. Investigation, ML, IK. Resources, ML, IK, AR, GW. Data curation, ML, IK. Writing – original draft preparation, ML. Writing – review and editing, ML, IK, AR, and GW. Project administration, GW. Acquisition, GW. All authors contributed to the article and approved the submitted version.

## Supporting information


**Table S1.** Oligonucleotides utilized for gene amplification via RT‐PCR and qRT‐PCR were designed using PrimerBlast (NCBI) and synthesized by Eurofins (Eurofins MWG Synthesis, Germany).
**Table S2.** Differentially expressed transcripts (*P* < 0.05, log_2_FC >1.0) within the proliferation‐related gene set in +CEACAM1‐4L and + CEACAM1‐4S MCF‐7 cells compared with VeCo, identified by RNA‐Seq.
**Fig. S1.** CEACAM1 in breast cancer tissues and lymph node metastasis.
**Fig. S2.** Lack of association between CEACAM1 expression patterns and ER, PR, or HER2 expression in breast cancer tissues.
**Fig S3.** Quantification of Ki‐67–positive cells in cell block immunocytochemistry images.

## Data Availability

The datasets generated and analyzed during the present study are included within the article and its Supplementary Information. Researchers interested in accessing the raw data may contact the corresponding authors via e‐mail. The datasets will be made available from the corresponding author upon reasonable request.
