# Supplementary material for: CEACAM1 participation in breast cancer progression
Source: Mol Oncol. 2026 Jul 10:10.1002/1878-0261.70306. Online ahead of print. doi: 10.1002/1878-0261.70306 (PMC13398363; doi:10.1002/1878-0261.70306)
Supplement: Supplementary file 1 — Table S1. Oligonucleotides utilized for gene amplification via RT‐PCR and qRT‐PCR were designed using PrimerBlast (NCBI) and synthesized by Eurofins (Eurofins MWG Synthesis, Germany). Table S2. Differentially expressed transcripts (P < 0.05, log2FC >1.0) within the proliferation‐related gene set in +CEACAM1‐4L and + CEACAM1‐4S MCF‐7 cells compared with VeCo, identified by RNA‐Seq. Fig. S1. CEACAM1 in breast cancer tissues and lymph node metastasis. Fig. S2. Lack of association between CEACAM1 expression patterns and ER, PR, or HER2 expression in breast cancer tissues. Fig S3. Quantification of Ki‐67–positive cells in cell block immunocytochemistry images. [file MOL2-9999-0-s001.docx]

**Supporting information**

**Suppl. Table S1:** Oligonucleotides utilized for gene amplification via RT-PCR and qRT-PCR were designed using PrimerBlast (NCBI) and synthesized by Eurofins (Eurofins MWG Synthesis, Germany).

| **Gene** | **Sequence (5`🡪 3`) Forward** | **Sequence (3`🡪 5`) Reverse** |
| --- | --- | --- |
| ***ACTB*** | GCTCGTCGTCGACAACGGCTC | CAAACATGATCTGGGTCATCTTCTC |
| ***GAPDH*** | TCAAGGCTGAGAACGGGAAG | TGGACTCCACGTACTCA |
| ***18S*** | CTACCACATCCAAGGAAGCA | TTTTTCGTCACTACCTCCCCG |
| ***CEACAM1-4S*** | AGACGATCATAGTCACTGAGCT | GGAGTGGTCCTGAGCTGC |
| ***CEACAM1-4L*** | AGACGATCATAGTCACTGAGCT | TGGAGTGGTCCTGAGTGTG |
| ***STAT1*** | GTTATGGGACCGCACCTTCA | AGTGAACTGGACCCCTGTCT |
| ***AREG*** | TGATACTCGGCTCAGGCCAT | CTCCCGAGGACGGTTCACTA |
| ***ETV4*** | CTTCGCCTACGACTCAGATGT | GGTTTCTCATAGCCATAGCCCA |
| ***ERBB4*** | CCTTTGTTATGCAGACACCATTCA | GGCAACGTCCACATCCTGA |
| ***MKI67*** | CTGCTTGTTTGGAAGGGGTATTG | ATTGCCTCCTGCTCATGGATT |
| ***RB1*** | GACACAACCCAGCAGTTCGATA | GAGCAACATGGGAGGTGAGAG |
| ***THBS1*** | AGAAGGACTCTGACGGCGA | GATGTCCCTTTGGGGTCCAG |

**Suppl. Table S2:** Differentially expressed transcripts (p < 0.05, log₂FC > 1.0) within the proliferation-related gene set in +CEACAM1-4L and +CEACAM1-4S MCF-7 cells compared with VeCo, identified by RNA-Seq.

| **+CEACAM1-4L *vs* VeCo** | | | | | | | | |
| --- | --- | --- | --- | --- | --- | --- | --- | --- |
| *HTRA1* | *FST* | *IGFBP5* | *HMOX1* | *GJA1* | *PGR* | *EGR3* | *OSR2* | *RB1* |
| *CCND2* | *TGFB2* | *ERRFI1* | *AREG* | *NUPR1* | *THBS1* | *APOA1* | *CLDN1* | *ETV4* |
| *NR4A1* | *FGF18* | *AGAP2* | *ERBB4* | *STAT1* |  |  |  |  |
| **+CEACAM1-4S *vs* VeCo** | | | | | | | | |
| *NR4A1* | *IGFBP5* | *NUPR1* | *PGR* | *ERBB4* | *FST* | *PTPRK* | *JAK2* | *STAT6* |

**
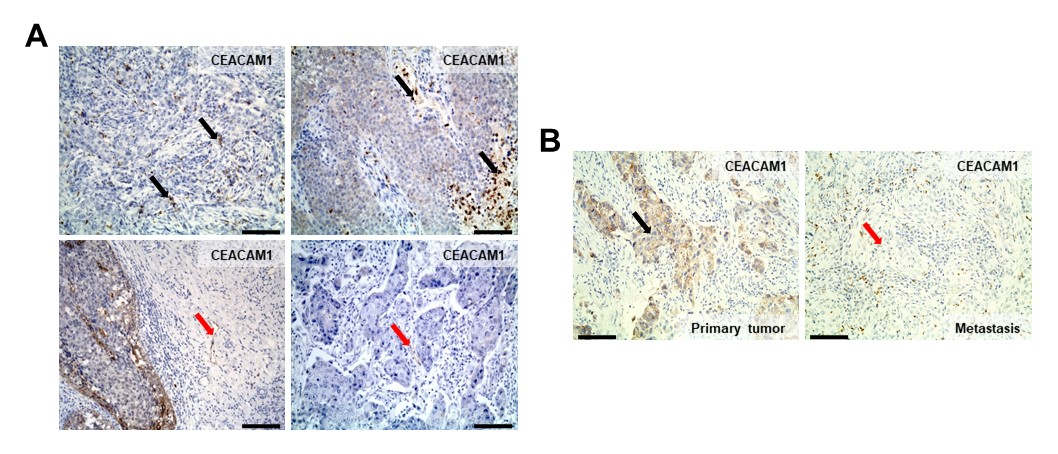
**

**Suppl. Figure 1. CEACAM1 in breast cancer tissues and lymph node metastasis.** **(A)** The figures show CEACAM1-positive leukocytes (indicated by black arrows) and CEACAM1 expression in the endothelium of isolated vessels (indicated by red arrows) within ВС tissue. Scale bar = 100 µm. **(B)** A case of breast cancer with uniform membranous and cytoplasmic distribution of CEACAM1 (indicated by black arrow) in the primary tumor and an absence of CEACAM1 (indicated by red arrow) in its lymph node metastasis. Scale bar = 100 µm.


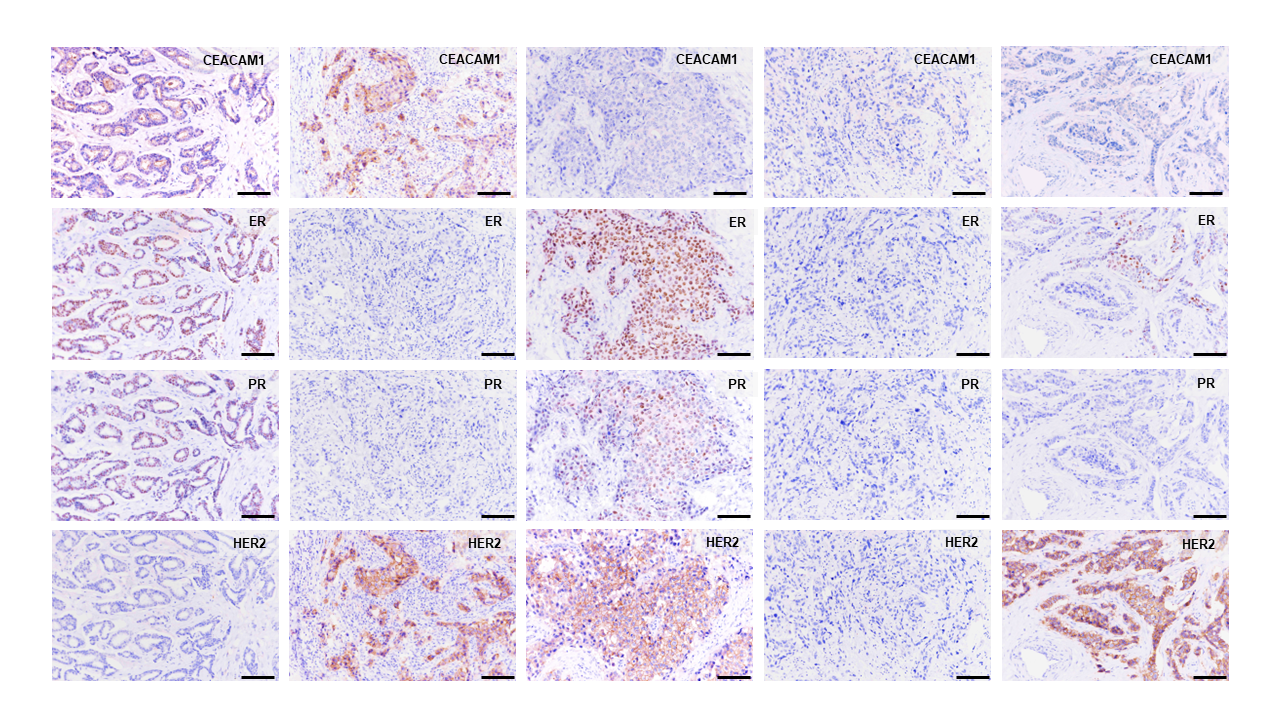


**Suppl. Figure 2. Lack of association between CEACAM1 expression patterns and ER, PR, or HER2 expression in breast cancer tissues.** Representative serial sections of breast cancer tissues demonstrating variable expression patterns of ER, PR, and HER2 independently of CEACAM1 expression status. Columns 1-2 show tumors with positive CEACAM1 expression exhibiting different ER/PR/HER2 expression profiles, whereas columns 3-5 represent CEACAM1-negative tumors with similarly heterogeneous receptor expression patterns. These findings illustrate that variability in CEACAM1 expression and localization is not accompanied by corresponding changes in ER, PR, or HER2 expression. Scale bar = 100 µm.


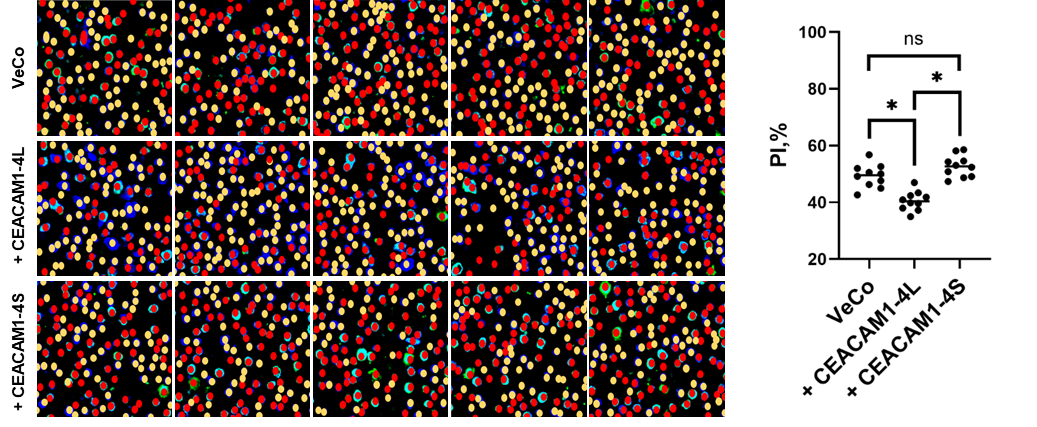


**Suppl. Figure 3. Quantification of Ki-67–positive cells in cell block immunocytochemistry images.** Representative 150 × 150 µm regions are shown for VeCo, +CEACAM1-4L, and +CEACAM1-4S. Ki-67–positive cells are marked with red dots, negative cells with yellow dots. The graph shows the proliferative index as mean percentage of Ki-67–positive cells; Student’s t-test, ns= not significant; *p < 0.05. n=10.
